# Letter to the Editor

**Published:** 1985-07

**Authors:** J. D. Davies

**Affiliations:** Bristol Royal Infirmary


### Letter to the Editor

Sir, 20 May, 1985

What is the most common benign tumour?

We all know the most difficult question any doctor gets is often the simplest. Once, I was foolish enough to tell a Primary FRCS Course that the most common benign tumour was a basal cell papilloma of skin (otherwise known as seborrhoeic keratosis). Most such audiences are depressingly passive, being too busy writing down the gems in the lecture. Not this one! There was a near-riot. No, the most common benign tumour is a lipoma I was told firmly. Even a mere pathologist is not expected to rake out the contents of the operating theatre's gash cans. How can they know anything about this subject if they do not even get sent such things?

Now several years on, I still wonder. What is the most common benign tumour? No reference book that I know gives a credible answer.

Maybe the readers of this *Journal* know the answer. Do please let us all know. I am sure that our Editor will allow the correction of a pathologist's ignorance. What is the experience in General Practice?

Yours faithfully,

J. D. Davies
Bristol Royal Infirmary

